# RNA-Chrom: a manually curated analytical database of RNA–chromatin interactome

**DOI:** 10.1093/database/baad025

**Published:** 2023-04-24

**Authors:** G K Ryabykh, S V Kuznetsov, Y D Korostelev, A I Sigorskikh, A A Zharikova, A A Mironov

**Affiliations:** Faculty of Bioengineering and Bioinformatics, Lomonosov Moscow State University, Leninskiye Gory, Moscow 119234, Russia; Kharkevich Institute for Information Transmission Problems RAS, Bolshoy Karetny per., Moscow 127051, Russia; Faculty of Bioengineering and Bioinformatics, Lomonosov Moscow State University, Leninskiye Gory, Moscow 119234, Russia; Kharkevich Institute for Information Transmission Problems RAS, Bolshoy Karetny per., Moscow 127051, Russia; Faculty of Bioengineering and Bioinformatics, Lomonosov Moscow State University, Leninskiye Gory, Moscow 119234, Russia; Faculty of Bioengineering and Bioinformatics, Lomonosov Moscow State University, Leninskiye Gory, Moscow 119234, Russia; Kharkevich Institute for Information Transmission Problems RAS, Bolshoy Karetny per., Moscow 127051, Russia; National Medical Research Center for Therapy and Preventive Medicine, Petroverigsky per., Moscow, 101000, Russia; Faculty of Bioengineering and Bioinformatics, Lomonosov Moscow State University, Leninskiye Gory, Moscow 119234, Russia; Kharkevich Institute for Information Transmission Problems RAS, Bolshoy Karetny per., Moscow 127051, Russia

## Abstract

Every year there is more and more evidence that non-coding RNAs play an important role in biological processes affecting various levels of organization of living systems: from the cellular (regulation of gene expression, remodeling and maintenance of chromatin structure, co-transcriptional suppression of transposons, splicing, post-transcriptional RNA modifications, etc.) to cell populations and even organismal ones (development, aging, cancer, cardiovascular and many other diseases). The development and creation of mutually complementary databases that will aggregate, unify and structure different types of data can help to reach the system level of studying non-coding RNAs. Here we present the RNA-Chrom manually curated analytical database, which contains the coordinates of billions of contacts of thousands of human and mouse RNAs with chromatin. Through the user-friendly web interface (https://rnachrom2.bioinf.fbb.msu.ru/), two approaches to the analysis of the RNA–chromatin interactome were implemented. Firstly, to find out whether the RNA of interest to a user contacts with chromatin, and if so, with which genes or DNA loci? Secondly, to find out which RNAs are in contact with the DNA locus of interest to a user (and probably participate in its regulation), and if there are such, what is the nature of their interaction? For a more detailed study of contact maps and their comparison with other data, the web interface allows a user to view them in the UCSC Genome Browser.

**Database URL** https://rnachrom2.bioinf.fbb.msu.ru/

**Key Points**

- Non-coding RNAs, while performing their function in the nucleus, play an important role in biological processes.
- The RNA-Chrom database currently stores all available genome-wide RNA–chromatin interactions data: 62 human and 125 mice experiments from 66 articles, totaling more than 5 billion RNA–DNA contacts.
- RNA-Chrom database contains not only the data processed with standardized protocol but also the comprehensive experiments metadata.
- RNA-Chrom provides a user-friendly web interface. Two types of the data analysis (‘from RNA’ and ‘from DNA’) can be performed.
- RNA-Chrom can be an important resource that will allow researchers to reach a more systematic level of work with the RNA–chromatin interactome and will promote to expand the understanding of the biological role of non-coding RNAs in a variety of processes.

## Introduction

Back in the 1960s, it was found that a large amount of different RNAs are associated with chromatin (1, 2). However, it remains unknown what kind of RNAs they are, which chromatin loci they prefer to interact with and what function they perform there. Much later, mainly using molecular biochemical methods, the functions of some non-coding RNAs were determined: XIST, which is responsible for X-chromosome dosage compensation in mammals (3), and Kcnq1ot1, which is involved in imprinting (4), and others. With the development of new methods, especially those involving a high-throughput sequencing step, more and more is becoming known about new chromatin-associated RNAs and their mechanisms of action.

For example, roX1 and roX2 RNAs are responsible for X-chromosome dosage compensation in *Drosophila*. There are also a number of regulatory RNAs (XIST, HOTAIR, MEG3, Paupar, ANRIL, TERRA, SRA, etc.) that affect gene expression by attracting chromatin modifiers such as TrxG and PRC1/PRC2 to certain loci. Another example is MALAT1 and NEAT1, which are associated with such nuclear structures as speckles and paraspeckles and regulate gene expression. Moreover, Firre can serve as a local organizing factor to ensure a topological proximity of trans-sites and its genomic locus; lnc-NR2F1 is involved in neurogenesis; Bloodlinc is involved in erythropoiesis; and DACOR1 interacts with maintaining DNA methyltransferase 1 and is expressed at a higher level in normal cells of the healthy colon and at a lower level in colon cancer cell clones. Many more biological examples of the interaction of RNA with chromatin can be cited (5).

To identify DNA loci contacted by non-coding RNAs, a number of experimental methods have been developed that can be divided into two groups. The first group of methods (RAP (6), CHART-seq (7), ChIRP-seq (8), dChIRP-seq (9), ChOP-seq (10) and CHIRT-seq (11)) solves the problem of finding contacts of a predetermined RNA. We will call this group of methods ‘one RNA to all DNA loci’ or ‘one-to-all’. The main idea of all these methods is as follows. Cells are fixed, resulting in covalent crosslinking of macromolecules. Next, DNA is fragmented, which results in a mixture of various complexes, including the RNA of interest with genomic DNA. Biotinylated oligonucleotides complementary to RNA of interest are used to isolate specific complexes. After isolation of the complexes with the target RNA on the streptavidin beads, the complexes are cleaved, the protein fraction is removed and the DNA is sequenced. It is assumed that the target RNA interacts with these DNA loci. The disadvantage of this approach is that the target RNA must be determined in advance (5).

**Figure 1.**
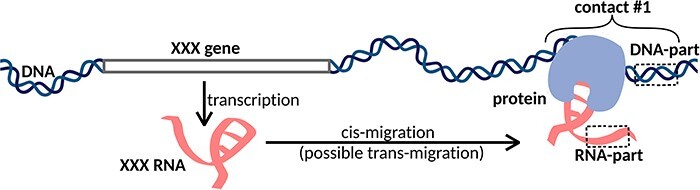
XXX RNA interacts with the DNA locus and forms contact #1. In the case of one-to-all methods, we see only the DNA-parts of the contacts, while in the case of all-to-all methods, we see both DNA-parts and RNA-parts of the contacts. Cis- and trans-migration is the migration of RNA within and outside the parent chromosome, respectively.

Another group of methods is designed to search for all possible DNA and RNA contacts in a cell (MARGI (12), GRID-seq (13–15), ChAR-seq (16), iMARGI (17, 18), RADICL-seq (19) and Red-C (20)). We will call this group of methods ‘all RNAs to all DNA loci’ or ‘all-to-all’. The main idea of this approach is that after cell fixation and DNA fragmentation, proximity ligation is carried out using a specially designed bivalent biotinylated bridge. After ligation of the bridge to RNA and then to DNA and reverse transcription, the chimeric constructs are sequenced. The result is a set of contacts of various RNAs and DNA loci (Figure 1). The disadvantage of this approach is that a large number of reads are needed to obtain reasonable data (5).

Many databases that facilitate the system level in the study of the non-coding RNAs action mechanisms are available (21). They can be roughly divided into ‘general’ databases, which aggregate a variety of non-coding RNA data (e.g. NONCODEV5 (22)), and ‘highly specialized’ ones, which focus either on a biological process (e.g. cancer — Cancer lncRNA Census (23)) or on a biological system (e.g. the cardiovascular system (24)) or on a specific type of data (e.g. histone modification and transcriptome data — HiMoRNA (25)).

In the case of the RNA–chromatin interactions, there is no database that is highly specialized on this type of data. On the other hand, there are a number of general databases with a web interface, which, among other things, contain collections of RNA–DNA contacts: RNAInter (26) and LnChrom (27) (unfortunately, the LnChrom has not been available for more than a year). RNAInter is the most comprehensive resource on the RNA interactome. An elementary object of this database is an RNA contact confirmed in one or another experiment, for which confidence score is calculated. However, this resource contains a small number of genome-wide experiments on RNA–chromatin contacts; in particular, apart from MARGI, there are no all-to-all data.

Comparative analysis of the RNA–chromatin interactome is of great scientific interest. To solve this problem, we have developed a highly specialized analytical database (https://rnachrom2.bioinf.fbb.msu.ru/) that contains all available genome-wide RNA–DNA interactions data. Since there is no standard protocol for processing these data, it is difficult to conduct a comparative analysis of RNA–chromatin contacts. Here we have standardized the data processing protocol and implemented it starting with raw reads. The RNA-Chrom database allows a user not only to download data processed by a single protocol but also to perform various methods of data analysis and comparison in real time. It is also possible to view contact maps in the UCSC Genome Browser (28) to study them in more detail and compare them with other data, such as DNA methylation data.

## Materials and methods

### General scheme of a web application

‘Front-end’ was developed using ‘Node.js’ (https://nodejs.org/en/) (an asynchronous event-driven JavaScript runtime), ‘React.js’ (https://reactjs.org/) and ‘Redux’ (https://github.com/reduxjs/redux) libraries and implemented as a ‘Single Page Application’. The ‘Material-UI V4’ (https://mui.com) library was taken as the basis for the web interface elements, and the ‘Plotly JavaScript Open Source Graphing Library’ (https://plot.ly/javascript/) was taken to create interactive plots. ‘Back-end’ was implemented using the Python web microframework ‘Quart’ (https://pgjones.gitlab.io/quart/) as it supports asynchronous database requests. The database itself, which stores RNA–chromatin contacts, was created on the basis of ‘ClickHouse’ (https://clickhouse.com/) (the Open Source OLAP database management system), due to which a user’s waiting time for any of their analytical requests was reduced to seconds.

### Extraction of the RNA–chromatin interactions data

Since the first articles with all-to-all methods appeared only in 2017, it was not difficult to find data corresponding to them. Things were quite different with one-to-all data. We first searched for them in Gene Expression Omnibus (http://www.ncbi.nlm.nih.gov/geo/) using the keywords ‘RAP-seq’, ‘CHART-seq’, ‘ChIRP-seq’, ‘dChIRP-seq’, ‘ChOP-seq’ and ‘CHIRT-seq’ and taking into account only human and mouse data sets. Then we went as far as possible through articles that referred to the main one-to-all methods (RAP (6), CHART-seq (7), ChIRP-seq (8), dChIRP-seq (9), ChOP-seq (10) and CHIRT-seq (11)). Surprisingly, we found a large number of publications that used one-to-all methods, but there were no publicly available data. Only one author responded to our request and provided us with RAP-data for Firre RNA (29). A total of 66 articles were found that had data in the public domain.

### Universal data processing protocol

**Figure 2.**
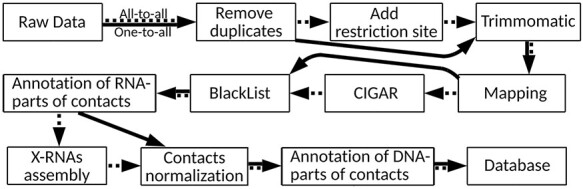
RNA–chromatin interactions data processing protocol. Dotted arrows correspond to all-to-all data processing steps and solid arrows are related to one-to-all data.

There are many approaches to RNA–chromatin interactions data processing, but each of them is tailored to the data obtained by a certain experimental method. Since it is necessary to use a single protocol for data unification and further comparative analysis, the authors of the LnChrom database used the protocol from the ChIRP article (8). Our database contains both all-to-all and one-to-all data, and we will base on the protocol applied in the Red-C experiment (20) (Figure 2), the details of which are disclosed in Supplementary Text 1.

**Table 1.** Human gene annotations (only from canonical chromosomes)

Annotations	Source	Number of genes
gencode	GENCODE v35 (34)	60 619
vlinc	article (35)	2762
GB_snomirna	UCSC Genome Browser^1^	2320
GB_trna	UCSC Genome Browser^2^	629
GB_repM	UCSC Genome Browser^3^	11 408
from_article	articles (36–38)	3
Xrna_human	assembled^4^ StringTie (39)	155 127
RNA-Chrom DB	articles (40–43)	2

^1^ wgRna table.

^2^ tRNAs table.

^3^ rmsk table.

^4^ Used data from the articles: GRID-seq (13), Red-C (20) and iMARGI (17, 18) (Supplementary Text 1).

**Table 2.** Mouse gene annotations (only from canonical chromosomes)

Annotations	Source	Number of genes
gencode	GENCODE M25 (34)	55 364
GB_trna	UCSC Genome Browser^1^	434
GB_repM	UCSC Genome Browser^2^	18 770
from_article	articles (44–47)	4
Xrna_mouse	assembled^3^ StringTie (39)	14 333
RNA-Chrom DB	articles^4^	9

^1^ tRNAs table.

^2^ rmsk table.

^3^ Used data from the articles: GRID-seq (13) and RADICL-seq (19) (Supplementary Text 1).

^4^ (11, 41, 48–56).

Raw data were downloaded from Gene Expression Omnibus and European Nucleotide Archive (https://www.ebi.ac.uk/ena/browser/home). Possible polymerase chain reaction duplicates were removed via FastUniq (30) and SeqKit (31) tools. ‘Add restriction site’ step was performed only for all-to-all data in strict accordance with the recommendations of the original articles. For all data, we used TRIMMOMATIC (v0.39) (32) for detection of low-quality position in each forward and reverse read. One-to-all and all-to-all data were mapped to the canonical chromosomes of the human and mouse genomes (GRCh38 and GRCm38 assemblies, respectively) with HISAT2 program (version 2.1) (33). After that, the orientations of the RNA-parts of the contacts were refined (Supplementary Text 2). It turned out that in the MARGI experiment (12), in most cases a random strand was read and the orientations of the RNA-parts were lost. Based on this finding, it was decided to exclude these data sets from any further analysis. To search for and process reads with splicing corresponding to RNA-parts of the contacts, the mapping information presented in the CIGAR field was analyzed. In turn, in accordance with the RADICL-seq protocol (19), those DNA-parts of the contacts that fell into the regions from the ENCODE BlackList were removed.

**Figure 3.**
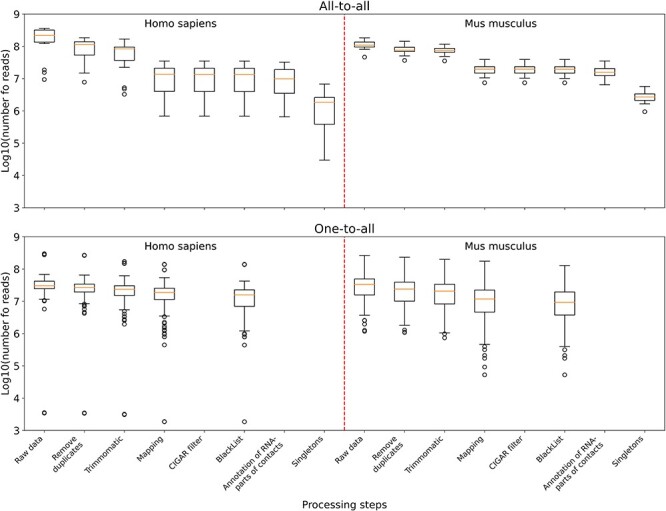
The distribution of the number of reads in the data sets left after the corresponding processing step and all the previous ones. Upper panel: boxplots plotted from all-to-all data, namely 17 human data sets and 18 mouse data sets. Lower panel: boxplots plotted from one-to-all data, namely 159 human data sets and 291 mouse data sets.

It is important for any contact to know the source gene. To do this, we collected the general gene annotation (Tables 1 and 2), balancing between its large size and the low representation of certain types of RNA. The clusters of unannotated RNA-parts of contacts were found and they were named X-RNAs. Only the contacts with RNA-parts that intersect the genes from the general annotation were added to the database. The others were named ‘Singletons’ and were not used. Having passed the data through all the previous steps of the protocol, we got the final number of contacts for each experiment. For all-to-all and one-to-all experiments, a background model was calculated, according to which each contact (in addition to the original or ‘Raw’ single value) was assigned a ‘Normalized’ value. Two additional normalizations were obtained for the one-to-all data: ‘Norm. & in peaks’ (background-normalized contacts crossing MACS2 peaks) and ‘Raw & in peaks’ (not-normalized contacts crossing MACS2 peaks). It is important to note that the median number of ‘reads in peaks’ for all experiments is 30 times less than the median number of reads that have passed all processing steps (Supplementary Figure 1). Thus, in our database there are four types of normalization. As a final step before loading the data into the database, we annotated DNA-parts of the contacts with genes and near-gene regions. To get the comparable characteristic of RNA contactability in all-to-all experiments, we introduced a ‘CPKM’ metrics — Contacts Per Kilobase of RNA length per Million filtered contacts in the experiment.

According to the summary statistics (Supplementary Table 1), for all-to-all data compared to one-to-all data, the largest number of reads is filtered out in the ‘Mapping’ step (Figure 3). This is because for all-to-all data, we require that the RNA- and DNA-parts of each contact are both correctly mapped; otherwise they will be filtered out. As for the one-to-all data, there are several data sets among them that are not credible. For example, GSM3073889 and GSM3073888 (human lincDUSP RNA) have <4000 raw reads and no MACS2 peaks. However, they are still in the database.

## Results

### Database statistics

In humans, 17 all-to-all data sets and 159 one-to-all data sets for 24 RNAs were collected (9 and 53 experiments in the database, respectively). In mice, 18 all-to-all data sets and 291 one-to-all data sets for 31 RNAs were collected (7 and 118 experiments in the database, respectively) (Supplementary Table 2). Since negative controls were not available for all experiments, they were not included in the universal data processing protocol and therefore in the database. In summary, the RNA-Chrom database contains more than 5 billion RNA–chromatin contacts and 232 870 human and 88 914 mouse genes. The general gene annotation includes public gene annotations (77 743 genes for human and 74 581 genes for mouse) as well as clusters of unannotated RNA-parts (155 127 X-RNAs for human and 14 333 X-RNAs for mouse), see ‘Universal data processing protocol’.

**Figure 4.**
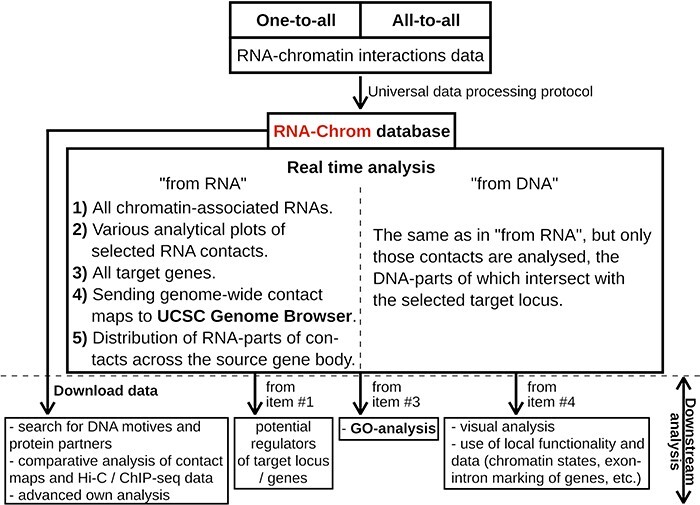
RNA-Chrom functionality and downstream analysis.

### The RNA-Chrom database functionality

**Figure 5.**
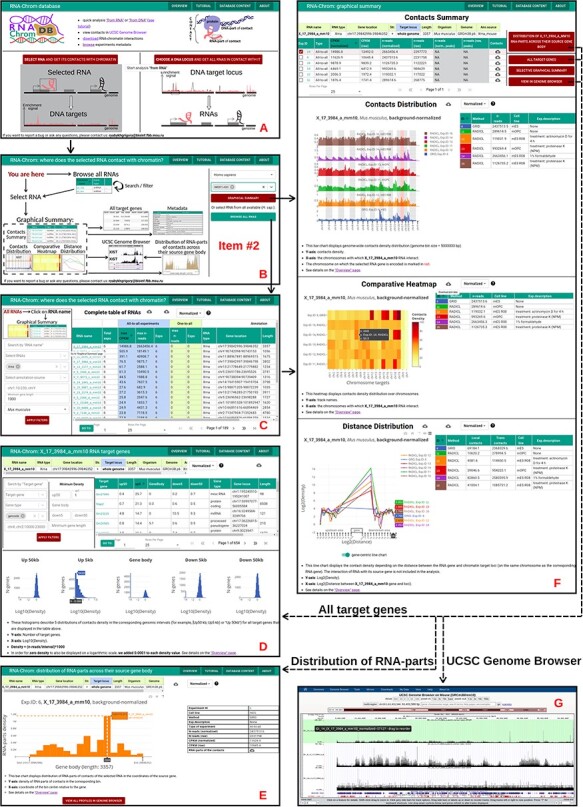
‘From RNA’ analysis for X_17_3984_a_mm10 RNA (*Mus musculus*). The arrows reflect the workflow. **A.** A user chooses ‘from RNA’ analysis. **B.** Then the user either enters the RNA name of interest in the ‘Select RNA’ field or presses ‘BROWSE ALL RNAS’ button. **C.** ‘Complete table of RNAs’. Here a user selects RNA for analysis. **D.** The ‘Graphical Summary’ page consists of the ‘Contacts Summary’ and three analytical plots. By selecting one or more contact maps of X_17_3984_a_mm10 RNA in the ‘Contacts Summary’ table, a user continues to analyze them by clicking on one of the four buttons located to the right of the table. **E.** ‘All target genes’ page displays the association of contacts with genes and their upstream and downstream regions. By applying several filters, a user downloads a list of target genes. From this point, the user can switch to ‘from DNA’ analysis. To do this, the user clicks on the target gene of interest. **F.** Distribution of X_17_3984_a_mm10 RNA-parts across their source gene body (may reflect the exon–intron structure, the multiple isoforms of the transcribed gene, etc.) A user can send distributions for all experiments to the UCSC Genome Browser for a more detailed study or download them. **G.** A user sends X_17_3984_a_mm10 contact maps (DNA-parts) to the UCSC Genome Browser if they want to view them in the higher resolution or visually match them to genomic annotations (gene sets, epigenetic marks, etc.) or data (ChIP-seq, Hi-C, etc.)

**Figure 6.**
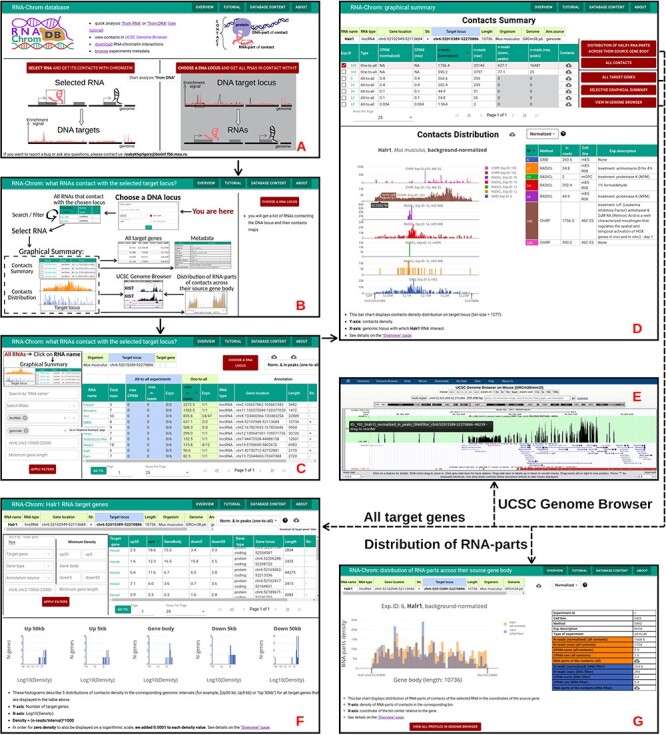
‘From DNA’ analysis for HoxA cluster (chr6:52 015 389-52 270 886, *M. musculus*). The arrows reflect the workflow. **A.** A user chooses ‘from DNA’ analysis and **B.** clicks on the ‘CHOOSE A DNA LOCUS’ button, selects the organism, enters the approximate coordinates of the HoxA cluster and clicks on the ‘APPLY’ button. **C.** All RNAs that contact with the chosen locus are presented in the table. A user applies filters if necessary. To go to the ‘Graphical Summary’ page, the user clicks on the RNA name of interest (for example, Halr1). **D.** The ‘Graphic Summary’ page is divided into two blocks: ‘Contacts Summary’ and ‘Contacts Distribution’. Five buttons located to the right of the ‘Contacts Summary’ table represent five options for further work with the Halr1 contacts. **E.** To get more details about the locus, a user sends contact maps to the UCSC Genome Browser. **F.** ‘All target genes’ page displays the association of contacts with genes located at the selected locus and their upstream and downstream regions. The gene list can be filtered in different ways. From this point, a user continues the analysis in the ‘from DNA’ way. **G.** ‘Distribution of Halr1 RNA-parts across their source gene body’ bar chart is plotted both for all contacts and for contacts with the target locus. These distributions can be downloaded or sent to the UCSC Genome Browser for a more detailed study.

With RNA-Chrom, a user can perform two types of analysis of the RNA–chromatin interactions data in real time that can transition into each other. We called the first type of analysis ‘from RNA’, since the first step is to select the RNA of interest. This analysis allows a user to answer the question ‘Where does the selected RNA contact chromatin?’. While the second type of analysis, ‘from DNA’, begins with the selection of the genomic locus of interest, and a user will receive an answer to the question ‘What RNAs contact with the selected target locus?’. These types of analysis include the following:

1. tables of RNAs contacting the entire genome (‘from RNA’ analysis) or the selected gene or locus (‘from DNA’ analysis), with the corresponding characteristics of their contactability (Figures 4, 5C, 6C);
2. tables of genes with which the selected RNA contacts directly or in the vicinity of 50 000 nucleotides (Figures 4, 5E, 6F);
3. three types of analytical plots (Figure 4):
  - contacts density distribution on target locus or on the whole genome (Figures 5D and 6D);
  - change in contact density depending on the distance between the RNA source gene and chromatin target loci (‘scaling’) (Figure 5D) and
  - distribution of RNA-parts of contacts across their source gene body (Figures 5F, 6G) and
4. the ability to view contact maps in the UCSC Genome Browser (Figures 4, 5G, 6E).

In addition, the RNA-Chrom database allows a user to download all pre-processed RNA–chromatin interactions data for a user’s own research or downstream analysis (Figure 4, ‘Downstream analysis’).

### Use case

#### ‘From RNA’ analysis

Using the web interface, a user can analyze the contacts of any RNA from our annotation. To perform ‘from RNA’ analysis, the following steps should be performed (Figure 5):

1. A user should choose ‘from RNA’ analysis on the start page (Figure 5A). The page ‘RNA-Chrom: where does the selected RNA contact with chromatin?’ will open in a new tab.
2. The next step depends on whether a user knows the RNA name they are interested in or wants to look at the ‘Complete table of RNAs’ (with contact metrics) and select one from it for further analysis.
  - For example, if a user is interested in NR2F1-AS1 RNA (*Homo sapiens*), they should enter the RNA name in the ‘Select RNA’ field, select the needed RNA in the drop-down list and then click on the ‘GRAPHICAL SUMMARY’ button (Figure 5B). The ‘Graphical Summary’ page will open in a new tab that will contain analytical interactive plots and various additional options for further analysis.
  - A user may click on the ‘BROWSE ALL RNAS’ button. At the ‘Complete table of RNAs’ different filters can be used, such as ‘Search by RNA name’, ‘Select RNA names’, ‘Select RNA types’, ‘Genomic loci’, etc. (Figure 5C). As an example, a user can fill the filters ‘Select RNA types’, ‘Minimum gene length’ and ‘Organism’ with the values ‘Xrna’, ‘1000’ and ‘*Mus musculus*’, respectively, and then click on the ‘APPLY FILTERS’ button. To go to the ‘Graphical Summary’ page, a user should click on the RNA name they are interested in (for example, X_17_3984_a_mm10, since this RNA has the largest ‘CPKM’).
3. The ‘Graphical Summary’ page (Figure 5D) consists of the ‘Contacts Summary’ and three analytical plots: ‘Contacts Distribution’, ‘Comparative Heatmap’ and ‘Distance Distribution’ (the details can be seen on the ‘Overview’ page).
4. In the ‘Contacts Summary’ table a user can choose, as an example, ‘Exp.ID: 14’ (RADICL, mES R08) and then click on one of the four buttons, for example, ‘ALL TARGET GENES’. The ‘All target genes’ page will open in a new tab.
5. From the ‘All target genes’ page (Figure 5E) a user can continue the analysis in ‘from DNA’ mode that will be described below and see what RNAs interact with this target gene or use filters to get a list of genes that can be downloaded for downstream analysis, such as Gene Ontology.

#### ‘From DNA’ analysis

This type of analysis allows a user to find all RNAs that contact with the selected gene or locus. To perform ‘from DNA’ analysis, the following steps should be taken (Figure 6):

1. A user should choose ‘from DNA’ analysis on the start page (Figure 6A). The page ‘RNA-Chrom: what RNAs contact with the selected target locus?’ will open in a new tab.
2. A user should click on the ‘CHOOSE A DNA LOCUS’ button located on the right side of the page (Figure 6B).
3. They can select the organism ‘*Mus musculus*’, enter the approximate coordinates of the HoxA cluster (chr6:52 015 389-52 270 886) and click on the ‘APPLY’ button. Another way is to select a locus by a gene name (Figure 6B).
4. After locus selection, a list of RNAs that contact with the selected locus appears. To work with this list, different filters can be used, and the list can be sorted in different ways (Figure 6C).
  - As an example, a user can fill the filters ‘Select RNA types’ and ‘Select annotation source’ with the values ‘lincRNA’ and ‘gencode’, respectively, and click on the ‘APPLY FILTERS’ button.
  - They can choose normalization ‘Norm. & in peaks (one-to-all)’ and sort the table by the column ‘max n-reads’ (one-to-all).
  - To go to the ‘Graphical Summary’ page, one should click on the RNA name they are interested in (for example, Halr1, since this RNA is at the top of the table and is also known to be involved in modulating HoxA induction (57)).
5. The ‘Graphical Summary’ page (Figure 6D) consists of the ‘Contacts Summary’ and the ‘Contacts Distribution’ analytical graph (the details can be seen on the ‘Overview’ page).
6. In the ‘Contacts Summary’ table one can choose, for example, ‘Exp.ID: 102’ (ChIRP, 46C ES, treatment: LIF withdrawal & 2uM RA—Day 1).
7. A user may choose normalization ‘Norm. & in peaks (one-to-all)’ and click on the ‘VIEW IN GENOME BROWSER’ button. UCSC Genome Browser will open in a new tab (Figure 6E).
8. If one clicks on the ‘ALL CONTACTS’ button, the ‘Graphical Summary’ page (Figure 6D) corresponding to the ‘from RNA’ analysis will open in a new tab. Here a user can continue the analysis in ‘from RNA’ mode.
9. A user can click on the ‘ALL TARGET GENES’ button, choose normalization ‘Norm. & in peaks (one-to-all)’ and fill the filter ‘Search by target gene’ with the value ‘hox’ (Figure 6F). Then they should click on the ‘APPLY FILTERS’ button. As expected, a user will see a lot of Hoxa genes. From the ‘All target genes’ page one can continue the analysis in ‘from DNA’ mode and see what RNAs interact with this target gene or download the list of genes for downstream analysis.

#### Content, metadata, overview and tutorial

The ‘Content’ page is a table with complete meta-information for all experiments from the RNA-Chrom database (Supplementary Table 2). Here a user can download data for each experiment (contacts with all normalizations, singletons, peaks, etc.). To find out detailed information on a particular experiment, a user should click on the corresponding ‘Exp.ID’ and go to the ‘Metadata’ page.

The ‘Metadata’ page contains all the metadata for a particular experiment, summary statistics on data processing, ‘Shares of different RNA types in the total number of contacts’ and ‘Distribution of the number of RNAs according to the number of contacts with the genome’ plots. A user can open the ‘Metadata’ page by clicking on the experiment ID everywhere the experiment ID appears, such as on the ‘Graphical Summary’, ‘All target genes’ and other pages.

The ‘Overview’ page describes the contents of the RNA-Chrom database and its functionality in detail, specifically what functions are available to a user, what formulas were used to preprocess data for plots and tables, and what information we can extract from those plots and tables.

The ‘Tutorial’ page contains ‘Basic’ and ‘Advanced’ examples of both ‘from RNA’ and ‘from DNA’ assays.

In order to go to the ‘Content’, ‘Overview’ or ‘Tutorial’ pages, a user should click on the corresponding buttons in the header of the website.

## Discussion

RNA-Chrom is the first manually curated database that contains a comprehensive collection of genome-wide human and mouse RNA–chromatin interactions data: 16 all-to-all experiments and 171 one-to-all experiments, totaling more than 5 billion RNA–chromatin contacts. We paid special attention to the outstanding procedure of data processing, and a user has the opportunity to evaluate the data quality. RNA-Chrom also provides a user-friendly web interface and two types of data analysis (‘from RNA’ and ‘from DNA’), which can be used for research. Throughout the analysis, a user has the opportunity to apply different filters, such as the type of normalization, the number of contacts of RNA with the whole genome or with the target locus, CPKM, the RNA type, the length of RNA source gene and others.

In order to determine the functional role of RNA in the corresponding DNA locus, additional genome-wide data and annotations are needed, for example, on the structure of chromatin, gene expression or the localization of DNA-binding and chromatin-modifying proteins. RNA-Chrom provides a variety of information about the interaction of RNA with chromatin, which can be used in a comparative analysis with other data or as a target for experimental refinements (Figure 4).

In the future, we plan to develop the database by adding new experiments and expanding the list of organisms, as well as to realize several additional normalization procedures that will take into account dependence of contact density on distance between the RNA source gene and chromatin target loci (‘scaling’), RNA expression level, etc. In addition to this, we are developing a special peak caller for RNA—DNA interactions data, which will be able to determine areas of statistically significant enrichment of the true signal compared to the background. As we can see, a significant fraction of reads was filtered out due to multiple mapping. We are going to develop approaches to the multiple mapping problem in these data. It is expected that the RNA-Chrom database will allow researchers to reach a more systematic level of work with the RNA–chromatin interactome, which will help to expand the understanding of the biological role of non-coding RNAs in a variety of processes.

## Supplementary Material

baad025_Supp
