# Correction to: RNA-Chrom: a manually curated analytical database of RNA–chromatin interactome

**DOI:** 10.1093/database/baad052

**Published:** 2023-07-01

This is a correction to: G K Ryabykh and others, RNA-Chrom: a manually curated analytical database of RNA–chromatin interactome, *Database*, Volume 2023, 2023, baad025, https://doi.org/10.1093/database/baad025

In the originally published version of this manuscript, the wrong files were published under two links for **Supplementary Data**. The correct files have now been supplied for both these links in the article.

